# Estimated Effects of Disinfection By-products on Preterm Birth in a Population Served by a Single Water Utility

**DOI:** 10.1289/ehp.9394

**Published:** 2006-10-04

**Authors:** Chad Lewis, Irwin H. Suffet, Katherine Hoggatt, Beate Ritz

**Affiliations:** 1 Environmental Science and Engineering Program, Department of Environmental Health Sciences, School of Public Health, University of California, Los Angeles, California, USA; 2 Department of Epidemiology, School of Public Health, University of Michigan, Michigan, USA; 3 Department of Epidemiology and; 4 Department of Environmental Health Sciences, School of Public Health, University of California, Los Angeles, California, USA

**Keywords:** disinfection by-products, environmental exposure, pregnancy second trimester, preterm birth, selective fetal loss, trihalomethanes, water

## Abstract

**Objectives:**

We evaluated the association between drinking-water disinfection by-products and preterm births using improved exposure assessment and more appropriate analysis methods than used in prior studies.

**Methods:**

During 1999–2001, vital record data were obtained for a large, racially diverse population residing in 27 Massachusetts communities that received drinking water from a single public utility. This water system was monitored weekly for total trihalomethanes (TTHM), and it maintained geographically stable total TTHM levels system-wide during the study period. We employed proportional hazards regression to examine the effects of trimester-specific and shorter-term peak exposures to TTHM in drinking water late in pregnancy on preterm births in 37,498 singletons.

**Results:**

For all women, our data suggested no more than a small increase, if any, in risk for delivering a preterm baby when exposed to ≥ 60 μg/L TTHM during the 4 weeks before birth [hazard ratio (HR) = 1.13; 95% confidence interval (CI), 0.95–1.35]. However, women who depended on a governmental source of payment for prenatal care were at increased risk when exposed at such levels late in gestation (HR = 1.39; 95% CI, 1.06–1.81). In contrast, exposure to high levels of TTHM during the second trimester and high exposure throughout pregnancy resulted in a 15–18% reduction in risk for preterm delivery in our population.

**Conclusions:**

This finding confirms previous reports of a negative association during the second trimester. Our data also suggested a possible positive association with shorter-term third-trimester TTHM exposure in mothers of lower socioeconomic status.

Disinfection of drinking water may result in hundreds of disinfection by-products (DBPs) (Richardson et al. 2002). Experimental, animal, and epidemiologic studies provide evidence that a number of these DBPs, including trihalomethanes, may be associated with adverse pregnancy outcomes (Balchak et al. 2000; Bove et al. 2002; Gemma et al. 2003; Goldman and Murr 2002; Plewa et al. 2004; Ward et al. 2000). However, epidemiologic studies exploring the effect of drinking water chlorination and total trihalomethane (TTHM) exposures on preterm birth in human populations have reported equivocal results. When simply comparing treatment type (i.e., chlorination vs. no chlorination) as a surrogate for DBP levels, several studies found increased risks of preterm birth (Kallen and Robert 2000; Kanitz et al. 1996; Tuthill et al. 1982; Yang 2004; Yang et al. 2000), but one reported negative associations, suggesting prolonged rather than shortened gestation in exposed subjects (Jaakkola et al. 2001). Studies that relied on quarterly TTHM measurements in water systems for exposure assessment also reported mixed results. A Massachusetts study found a small decrease in risk of preterm birth among women who experienced high second-trimester or total pregnancy exposure to TTHM, but no association was observed for third-trimester exposures (Wright et al. 2003). However, in a second study, the same authors found that third-trimester exposures apparently decreased the risk of delivering a preterm infant (Wright et al. 2004). A New Jersey study reported a slightly increased risk for preterm births with high average exposures to TTHM throughout pregnancy, but the effects were nonlinear (Bove et al. 1992). This group also estimated stronger effects on preterm births in women whose drinking water originated from surface rather than groundwater. Finally, studies conducted in Arizona, North Carolina, Iowa, Colorado, and Canada have not found associations between preterm births and elevated levels of TTHM ( Dodds et al. 1999; Gallagher et al. 1998; Hinckley et al. 2005b; Kramer et al. 1992; Savitz et al. 1995).

For water systems serving > 10,000 people in the United States, regulatory monitoring data consist of samples taken at four sites and typically collected quarterly; thus, these data may not accurately capture spatiotemporal variability in DBPs [U.S. Environmental Protection Agency (EPA) 1998]. Previous epidemiologic studies of preterm birth relying on such data for exposure assessment thus necessarily operated at a relatively crude time scale. In this study, we were able to improve the time scale by employing weekly monitoring data for TTHMs. In addition, we decreased heterogeneity in TTHM across water distribution systems by relying on only a single public utility serving multiple communities in Massachusetts. Specifically, the water treatment regimens employed by this utility minimized the geographic variability of TTHMs. The importance of such a feature for valid exposure assessment in epidemiologic studies is underscored by recent research to develop methods to identify utilities that have low spatial variability of DBP (Hinckley et al. 2005a).

Linking 2 years of birth data to weekly monitoring data for TTHMs enabled us to model the associations between shorter-term peak exposures and preterm births. Unlike most previous research, in our study we matched cases and controls closely on gestational age at exposure to address the influence of exposure timing—the existence of vulnerable windows late in pregnancy. Finally, our study population is diverse and large enough to allow us to examine the relation between preterm births and TTHM in the water supplied to homes for different racial/ethnic and socioeconomic groups.

## Materials and Methods

Data were obtained from the Registry of Vital Records and Statistics, Massachusetts Department of Public Health (Boston, MA). We abstracted 39,593 records of singletons conceived between the beginning of February 1999 and the end of February 2001 whose mothers, at the birth of their child, resided in a community served only by the utility studied. This 2-year period represented the time during which complete exposure during pregnancy could be ascertained for all preterm and term births. We restricted our analyses to infants between 32 and 45 gestational weeks with a birth weight between 500 and 5,000 g (*n* = 905 excluded). Furthermore, if the birth records did not provide information for one of the following variables, subjects were excluded from the analyses: sex, maternal age, marital status, race-ancestry, maternal education, parity, cigarette smoking, payment source, or a maternal disease factor (*n* = 1,190 excluded), leaving us with 37,498 births. Cases were defined as preterm birth or infants born at < 37 completed weeks of gestation, calculated from date of last menstrual period (LMP) provided in the birth file. Cases were matched to controls by gestational age; thus, controls were still unborn at the gestational time the preterm births occurred.

### Water quality data

We abstracted trihalomethane data from the Massachusetts Department of Environmental Protection (MDEP 2003) records for 27 communities receiving water from a single supplier. Trihalomethane concentrations were determined by U.S. EPA method 524.2 (Sung et al. 2000; U.S. EPA 1995) and consisted of chloroform, bromoform, bromodichloromethane, and dibromochloromethane. The sum of measurements for all constituents represents our measure of TTHM. The details of the water treatment and distribution are provided elsewhere (Lewis et al. 2006; Sung et al. 2000).

### Exposure assessment

We employed data on maternal residence at birth, gestational age, and environmental sampling to create a total TTHM exposure estimate for each gestational period. For each infant we calculated exposure measures of average TTHM for the first (gestational days 1–93), second (gestational days 94–186), and third trimester (gestational days 187–280), and 4, 2, and 1 weeks before birth for both cases and unborn controls matched by gestational age to each case. Relying on weekly TTHM samples, we calculated each mother’s average exposure for the aforementioned time frames for the maternal residence reported on the birth certificate. In the following, we consider TTHM exposures averaged over 1 week to 1 month, rather than a whole trimester or all of pregnancy, as “peak” exposures. Pregnancy length in days was calculated by multiplying the LMP gestational age, presented in weeks, by seven, thus assuming the value represented completed weeks of gestation (Lewis et al. 2006). The weekly average TTHM of four sampling sites was applied to 24 of 27 communities as described in our previous publication (Lewis et al. 2006). Initially we considered exposure categories in 10-μg/L increments, but it became necessary to collapse categories to improve estimation. While doing so, we tried to maintain consistency with our prior publication and cut points used in previous research (Lewis et al. 2006).

### Statistical methods

We employed risk sets (Breslow and Day 1987) to control for timing (gestational age) of exposure to TTHM during the third trimester and to hold the duration of exposure constant. For example, if a preterm birth occurred during the 32nd week of gestation, controls comprised all children still in the womb during week 32; thus, both cases and controls had a TTHM 4-week average exposure that spanned from week 28 to 31 of gestation. Indicator variables were used for infant sex, marital status (married, not married, and previously married within 300 days), adequacy of prenatal care (Kessner Index) (Kessner et al. 1973), maternal age (< 20, 20–29, 30–34, 35–39, or ≥ 40 years of age), maternal race/ethnicity (African American, Asian, Caucasian, Hispanic, or other), maternal education (< high school, high school degree, associate’s degree, bachelor’s degree, or postgraduate), interval since the previous live birth (≤ 12 months or > 12 months), maternal smoking during pregnancy (0, 1 to 5, 6 to 10, or ≥ 11 cigarettes/day), previous infant weighing > 4,000 g, previous preterm or small-for-gestational-age (SGA) infant, prenatal care source of payment (private, Healthy Start, or government), conception season (i.e., four periods of 3 months’ length), birth season, and community per capita income taken from U.S. Census Bureau data (< $23,000, $23,000 to < $26,000, $26,000 to < $33,000, or ≥ $33,000) (U.S. Census Bureau 2001). A single indicator variable was created to indicate the presence of one or more of the following maternal diseases during pregnancy: diabetes, eclampsia, hydramnios, chronic hypertension, lung disease, pregnancy-related hypertension, incompetent cervix, lupus, renal disease, uterine bleeding, and inappropriate weight gain/loss. Our final models contain all listed potential risk factors for preterm birth reported on birth certificates including 11 of 21 maternal diseases experienced during pregnancy (10 diseases did not influence results or were extremely rare). We conducted analyses stratified by some of our individual- and community-level indicators for socioeconomic status (SES) to examine effect measure modification. SES strata were defined as “low” if prenatal care source of payment was Healthy Start or government, per capita income was < $26,000, or maternal education was an associate’s degree or less; and defined as “high” if the payment source was private, community per capita income was > $26,000, or maternal education was a 4-year degree or more.

## Results

A total of 894 trihalomethane samples were abstracted for six monitoring sites from 1999 through 2001. The interquartile range of the monthly TTHM distributions was 59 μg/L (minimum = 28 μg/L; maximum = 87 μg/L). The timing and duration of the TTHM peaks varied each year, sometimes occurring as early as May or as late as August. The main component of TTHM in this water system was chloroform, contributing 83–93% of the TTHM (average, 89%) to the monthly average fraction. Bromodichloromethane ranged from not detected to 9 μg/L (75th percentile, 6.1 μg/L). Dibromochloromethane and bromoform were not detectable or were measured at very low levels (< 1 μg/L) (Lewis et al. 2006).

The mean birth weight of the 37,498 infants included in our analysis was 3,405 g. Among these infants, 2,813 (7.5%) were classified as preterm births. The distributions of major predictors and TTHM exposure estimates for preterm births are shown in Tables 1 and 2. In general, mothers who were ≤ 20 or > 40 years of age, African American or Hispanic, lacked adequate prenatal care, or suffered from a disease during pregnancy were more likely to deliver preterm. The percent of preterm births declined with increasing TTHM exposure during the second trimester.

### Preterm births

For all women, our data were consistent with a decrease in the risk of preterm births for high levels of TTHM (≥ 60 μg/L) exposure during the second trimester [hazard ratio (HR) = 0.82; 95% confidence interval (CI), 0.71–0.94] and also during the whole of pregnancy (HR = 0.85; 95% CI, 0.74–0.97). A negative trend was observed per 10-μg/L increase in TTHM for both exposure-averaging periods (Table 3).

In contrast, there was a weak association between previous-4-weeks high exposure to TTHM and preterm birth (HR = 1.13; 95% CI, 0.95–1.35) (Table 3), but the estimate was relatively imprecise. Results were similar for the highest TTHM levels averaged over only 2 weeks and 1 week before birth (HR 1.15; 95% CI, 0.97–1.35 2-week average; and HR 1.06; 95% CI, 0.92–1.23 1-week average) (data not shown).

When examining the influence of TTHM exposure by maternal race/ethnicity, we found that high exposures (≥ 60 μg/L TTHM) experienced during the second trimester were negatively associated with preterm birth, most strongly in African Americans (HR = 0.62; 95% CI, 0.46–0.84), possibly Asians (HR = 0.72; 95% CI, 0.44–1.20), and to a lesser degree in Caucasians (HR = 0.86; 95% CI, 0.71–1.05), but was not associated with preterm birth in Hispanic women (Table 3). Associations were also observed between previous-4-weeks medium and high exposure to TTHM and preterm birth among Hispanic women (Table 3). When we stratified our data by payment source as an indicator of maternal SES, negative trends and a decrease in risk at high levels of second-trimester exposure were observed for both low- and high-SES women (Table 4).

Preterm birth was also associated with high levels of exposure to TTHM (≥ 60 μg/L) experienced during the last 4 weeks before birth by women with a government source of payment for prenatal care (HR = 1.39; 95% CI, 1.06–1.81) (Table 4). Inclusion of an interaction term between TTHM exposure and source of prenatal care marginally improved the fit of the model (*p* = 0.12) (data not shown). The point estimates for low-SES mothers increased when the analyses were restricted to gestational ages < 36 weeks (HR = 1.49; 95% CI, 1.04–2.13) or < 35 weeks (HR = 1.67; 95% CI, 1.01–2.74) (data not shown). Increased risk of preterm birth was also associated with high TTHM during the last 4 weeks before birth in women categorized as having low per capita income (< $26,000) (HR = 1.19; 95% CI, 0.97–1.46) and in women with an associate’s degree or less (HR = 1.14; 95% CI, 0.91–1.41), but the estimated effect sizes were smaller than those for source of payment.

## Discussion

We relied on information from birth certificates and TTHM data sampled at high frequency to construct risk sets (Breslow and Day 1987) and derive gestational age–matched TTHM exposure measures for residents of Massachusetts communities served by a single public utility. Our results were consistent with those of an earlier Massachusetts study that reported a decrease in risk of preterm delivery when mothers experienced high exposures during the second trimester (Wright et al. 2003). However, our results were also consistent with a possible increase in risk of delivering preterm when TTHM levels were high 4 weeks before delivery in the third trimester for mothers who had to rely on a government source of payment for prenatal care. Equivocal and inconsistent results from previous epidemiologic research on TTHMs and preterm births may be attributable to several factors, including the lack of controlling for timing and duration of exposure among cases and controls when estimating third-trimester effects, a lack of examining susceptible subgroups, crude/biased exposure assessments, and lack of control for exposures received before the third trimester.

To date, no other published study has described an increased risk of preterm birth and TTHM exposure during the short period before delivery while matching on gestational age (Hinckley et al. 2005b). We explored 4-, 2-, and 1-week time windows of exposure before preterm delivery but found little difference between effect estimate sizes, suggesting that it is not a very short-term acute effect of exposure. We examined the potential impact of inaccurately assigned gestational age by shortening the gestational age for our case definition to 35 and 34 weeks and found that effect estimates were similar to those for 32–36 weeks, or even increased. The increased risk observed among Hispanic women lacks a dose–response pattern, and the diversity of the Massachusetts Hispanic population renders interpretation difficult; but research has shown demographic variables to be strongly predictive of use and consumption of water (Forssén et al. 2006). We did not evaluate an interaction term for ethnic group and TTHM. Although there was suggestion of an interaction between ethnic group and TTHM, our sample size was insufficient to estimate this interaction with precision, and our results were also consistent with effect homogeneity across ethnic groups.

Our negative effect estimates for the second trimester and pregnancy overall are consistent with research by Wright et al. (2003), who reported reduced risk for second trimester [odds ratio (OR) = 0.90; 95% CI, 0.79–1.03] and pregnancy average (OR = 0.90; 95% CI, 0.77–1.04) exposure > 80 μg/L TTHM compared with < 60 μg/L. The authors (Wright et al. 2003) also provide evidence of a positive trend with length of gestational age—higher TTHM exposure and increasing gestational length. Previously, we reported a second-trimester DBP association with fetal growth that may differ by race/ethnicity (Lewis et al. 2006). Thus, our current results suggest a mirror-image TTHM effect in opposite directions for growth retardation and preterm birth. A possible explanation might be that high exposure to TTHM during the second trimester may prolong pregnancy through mechanisms that may also restrict fetal growth and thus result in small babies born at term.

Recent research exploring the influence of cytokines on preterm and SGA birth may provide some support for this explanation (Engel et al. 2005). Polymorphisms in pro-inflammatory cytokines [i.e., tumor necrosis factor (TNF)] have been associated with preterm births (Crider et al. 2005; Engel et al. 2005), whereas polymorphisms in anti-inflammatory cytokines [i.e., interleukin (IL)-4] have been associated with SGA outcomes (Engel et al. 2005). In animals, both of these cytokines may be affected by exposure to carbon tetrachloride, a haloalkane similar to chloroform, and to phosgene, a metabolite of chloroform (Sciuto et al. 2003; Weber et al. 2003). Engel et al. (2005) outlined a conceptual model of how a perturbation in the balance of pro- and anti-inflammatory cytokines during pregnancy may determine the time of delivery and result in preterm or SGA birth. Specifically, these authors suggested that a decreased production of anti-inflammatory cytokines may increase the risk of spontaneous preterm birth but decrease the risk of SGA, whereas an increased production of anti-inflammatory cytokines may increase the risk of SGA (Engel et al. 2005). Elevated levels of both pro-inflammatory (TNF, IL-6, IL-8) and anti-inflammatory (IL-10; transforming growth factor) cytokines have also been identified in animals exposed to carbon tetrachloride (Weber et al. 2003). In addition, mice exposed to phosgene exhibited increased levels of pro-inflammatory cytokines IL-6, TNF-α, IL-1β, macrophage inflammatory protein-2, and anti-inflammatory cytokine IL-10, but decreased levels of IL-4 (Sciuto et al. 2003). It is therefore possible that timing of and weight at birth may depend on the balance of these cytokines. Exposure to DBPs may alter that balance depending on the baseline state of the mother (biologic and genetic susceptibility) and the timing of exposure.

However, our results could also reflect an effect of DBPs on fetal death. In our study, we focused on the association between DBPs and preterm birth among live-born infants only. If high second-trimester levels of DBPs were to cause fetal death among vulnerable conceptuses—that is, those conceptuses who were at greatest risk for preterm birth due to other factors—then among survivors DBPs might appear to be inversely associated with preterm birth. This phenomenon reflects a type of selection bias inherent in studies of birth outcomes (Hernan et al. 2002, 2004). We examined the plausibility of this selection bias by estimating whether there was a sufficient number of highly exposed (TTHM ≥ 60 μg/L) fetal deaths to modify observed ORs of preterm births. Using aggregate monthly data, we estimated the range of fetal deaths that would have experienced an elevated 4-week average TTHM exposure (i.e., ≥ 60 μg/L and ≥ 70 μg/L) during the month of death as well as lags of 1, 2, and 3 months before the death. This approach resulted in a range of 71–103 highly exposed fetal deaths during the study period. When these numbers (71 fetal deaths) were added to the number of highly exposed preterm births, the OR estimate changed from negative (OR = 0.90; 95% CI, 0.81–1.00) to null (OR = 0.98; 95% CI, 0.89–1.09). Therefore, selection bias cannot be ruled out as a possible explanation for the negative associations observed here and in previous research, although recent research suggests that DBPs in the range commonly encountered in the United States may not affect survival (Savitz et al. 2006).

SES is a complex concept that has been associated with a range of diseases. We explored individual- and community-level indicators of SES and found that source of payment for prenatal care and less clearly a combination of per capita income at the community level and maternal education as an indicator of SES may modify the risk of preterm birth due to shorter-term TTHM exposures [high TTHM exposures (≥ 60 μg/L)] during the last 4 weeks before birth: low SES: HR = 1.25; 95% CI, 0.94–1.65; high SES: HR = 0.97; 95% CI, 0.65–1.44; government source of prenatal care: HR = 1.39; 95% CI, 1.06–1.81; and private source of payment for prenatal care: HR = 1.00; 95% CI, 0.79–1.25. It is possible that the indicators of low SES serve as a proxy for an increased biologic susceptibility to toxicants or reflects actual differences in exposure by SES. Low-SES women may be more susceptible to TTHM exposures during the third trimester (i.e., shortly before normal delivery) because they already experience higher levels of other environmental or social stressors, increasing their vulnerability in general (Perera et al. 2004). In addition, low-SES women may not receive the same medical attention as other women when they experience signs of preterm labor and thus may be at increased risk of preterm birth. Compared with mothers with private [i.e., HMO (health management organization)] sources of payment for prenatal care, the mothers with a government source of pay for prenatal care were young, had a high school education or less, and lacked adequate prenatal care based on the Kessner Index. We do not believe that SES differences resulted in differences of TTHM concentrations at the residence because TTHMs exhibited little intrasystem variability during our study period in general.

Our models included previous trimester exposure—HRs for prior 4 weeks exposures were adjusted for first- and second-trimester TTHM exposure. When we estimated effects for 4 weeks before birth during the third trimester ignoring previous exposures, we did not find any effects at high exposure (i.e., TTHM ≥ 60 μg/L): HR = 0.99; 95% CI, 0.87–1.14; government source of prenatal care: HR = 1.14; 95% CI, 0.92–1.14; private source of payment for prenatal care: HR = 0.92; 95% CI, 0.77–1.09. Thus, effect estimates for TTHM exposure during the previous 4 weeks before birth may be confounded by previous trimester exposures. However, second-trimester effects continued to be negative at high-TTHM exposure whether or not we adjusted for first-trimester exposures (HR = 0.86; 95% CI, 0.76–0.97). Adjusting for previous trimester exposure assumes that exposures and health impacts from conception through birth are part of a continuous process and that exposures experienced during an earlier period may alter the effect these exposures have during a later period (Selevan and Lemasters 1987; Weinberg and Wilcox 1998).

We were able to use more frequently sampled monitoring data in communities served by one utility during a period in which treatment regimens and raw water quality created seasonally variable but spatially stable intrasystem TTHMs, as described previously (Lewis et al. 2006). Not only does the use of weekly monitoring data capture temporal peaks in TTHM better than quarterly data, but our approach resulted in studying a population’s exposure over time rather than comparing populations from different communities with varying exposure levels to each other. Thus, we reduced the risk of confounding by geographic and demographic risk factors, because there are far fewer time-varying risk factors we are aware of that might correlate with TTHM levels and thus confound our risk estimates. Improved exposure assessment also is likely to reduce the potential for exposure misclassification.

We used maternal address at birth to assign exposures, assuming that this was the residence for the entire pregnancy. Research indicated that a substantial number (25%) of women are likely to move between conception and delivery, although most of these women move within the county of residence (Khoury et al. 1988; Shaw and Malcoe 1992). We do not believe that a large percentage of women moved out of the water service area we studied because 24 communities in our study area were adjacent to each other, as described elsewhere (Lewis et al. 2006). For women who move into the water service area during pregnancy, we would expect that moving is not affected by (subsequent) outcome status, and therefore estimates would likely be affected by nondifferential exposure misclassification. However, the direction of bias is difficult to predict without a quantitative assessment.

As in previous studies, we were unable to distinguish between various pathways of exposure. Substantial exposure to the volatile portion of DBPs might occur through dermal contact and inhalation during showering, bathing, swimming, and hand dishwashing but we were unable to measure the contribution of such exposures in this study (Nuckols et al. 2005).

There is a possibility of residual confounding in our study because of lack of information on selected risk factors such as maternal nutrition, job-related risk factors, maternal infection rates, indoor/outdoor air pollution, and other water contaminants. However, these risk factors would have to be associated with TTHM exposure levels to act as confounders—that is, they would have to be varying over time in concert with the variation of TTHM levels. Although air pollution is time varying and could be a potential confounder, the air pollutants most often linked to preterm birth in the literature are combustion by-products (i.e., sulfur dioxide) and would be expected to be highest in winter (Maisonet et al. 2001). This is anticyclical to TTHM levels, which tend to increase with water’s ultraviolet-254 absorbance and temperature (Sung et al. 2000). Thus, we would expect negative confounding of our associations, if any. The same would be true for maternal respiratory infections, whereas nutrition and job-related factors are likely not seasonal in the population studied.

## Conclusion

In contrast to previous studies, we used risk-set matching in our case–control study of preterm births and obtained results consistent with a negative association for second-trimester TTHM exposure and a possible positive association with shorter-term third-trimester TTHM exposure in mothers of low SES. Future research on preterm births and DBPs should employ survival analysis methods when estimating third-trimester effects, adjust for exposure received in all three trimesters, and if at all possible investigate risk in susceptible subgroups—that is, investigate the influence of race/ethnicity and SES in conjunction with exposures on the outcomes.

## Figures and Tables

**Table 1:** Demographic characteristics of mothers and their singleton infants in selected Massachusetts communities served by a single surface–supplied drinking water utility, 1999–2001.

Characteristic	Study population [no. (%)]	Percent preterm (*n* = 2,813)
Total	37,498 (100)	7.5
Sex		
Male	19,219 (51.3)	7.8
Female	18,279 (48.7)	7.1
Age (years)		
< 20	2,136 (5.7)	9.2
20 to 29	13,937 (37.2)	7.7
30 to 34	12,671 (33.8)	6.9
35 to 39	7,144 (19.1)	7.5
≥ 40	1,610 (4.3)	8.3
Race		
Caucasian	21,945 (58.5)	6.5
African American	5,846 (15.6)	10.4
Hispanic	5,957 (15.9)	9.1
Asian	3,716 (9.9)	6.3
Other	34 (0.1)	8.8
Prenatal care^a^		
Adequate	27,941 (74.5)	7.0
Intermediate	4,995 (13.3)	9.9
Inadequate	992 (2.6)	10.7
No prenatal care	48 (0.1)	25.0
Unknown	3,522 (9.4)	7.2
Parity		
1	18,235 (48.6)	7.9
2 to 3	16,883 (45.0)	6.8
4 to 5	2,069 (5.5)	9.5
≥ 6	311 (0.8)	9.3
Maternal disease factor present		
None	31,556 (84.2)	6.5
≥ 1	5,942 (15.8)	12.9
Prenatal care payment source		
Private or HMO	25,708 (68.6)	6.6
Government or Healthy Start	11,790 (31.4)	9.6

a Kessner Index (Kessner et al. 1973)

**Table 2:** Study population and percent preterm births by TTHM (μg/L) level averaged over different gestational periods.

TTHM (μg/L)	Study population [no. (%)]	Percent preterm (*n* = 2,813)
Total	37,498 (100)	7.5
First trimester		
< 40	10,831 (28.9)	7.2
40 to < 60	17,588 (46.9)	7.6
≥ 60	9,079 (24.2)	7.7
Second trimester		
< 40	10,604 (28.3)	7.9
40 to < 60	16,059 (42.8)	7.5
≥ 60	10,835 (28.9)	7.2
Previous 4-week average		
< 40	10,300 (27.5)	8.2
40 to < 60	13,031 (34.8)	7.1
≥ 60	14,167 (37.8)	7.4
Pregnancy average		
< 40	7,222 (19.3)	8.2
40 to < 60	21,399 (57.1)	7.3
≥ 60	8,877 (23.7)	7.4

**Table 3:** Adjusted^a^ HRs (95% CIs) for preterm birth and gestational age-specific TTHM exposure averages by race and ancestry.

TTHM (μg/L)	1st Trimester	2nd Trimester	4-Week risk sets	Pregnancy average
All races (*n* = 2,813)				
< 40	1.00	1.00	1.00	1.00
40 to < 60	1.02 (0.92–1.13)	0.87 (0.77–0.99)	1.00 (0.87–1.15)	0.92 (0.82–1.02)
≥ 60	1.00 (0.88–1.14)	0.82 (0.71–0.94)	1.13 (0.95–1.35)	0.85 (0.74–0.97)
Per 10 μg/L	0.98 (0.95–1.02)	0.95 (0.92–0.99)	1.01 (0.97–1.06)	0.95 (0.91–0.99)
Caucasians (*n* = 1,429)				
< 40	1.00	1.00	1.00	1.00
40 to < 60	0.97 (0.84–1.12)	0.93 (0.77–1.11)	0.97 (0.79–1.18)	0.92 (0.79–1.06)
≥ 60	1.04 (0.87–1.24)	0.86 (0.71–1.05)	1.14 (0.90–1.45)	0.81 (0.67–0.99)
Per 10 μg/L	0.99 (0.94–1.05)	0.95 (0.90–1.00)	1.04 (0.97–1.10)	0.96 (0.91–1.03)
African American (*n* = 607)				
< 40	1.00	1.00	1.00	1.00
40 to < 60	1.27 (1.00–1.60)	0.73 (0.56–0.96)	0.78 (0.57–1.07)	0.95 (0.74–1.20)
≥ 60	1.13 (0.84–1.52)	0.62 (0.46–0.84)	1.00 (0.69–1.45)	0.77 (0.57–1.05)
Per 10 μg/L	1.01 (0.93–1.10)	0.89 (0.82–0.96)	0.98 (0.89–1.08)	0.90 (0.81–0.99)
Hispanic (*n* = 541)				
< 40	1.00	1.00	1.00	1.00
40 to < 60	0.97 (0.75–1.24)	1.07 (0.80–1.43)	1.59 (1.15–2.21)	0.87 (0.68–1.12)
≥ 60	0.86 (0.63–1.17)	1.07 (0.78–1.46)	1.63 (1.09–2.43)	1.00 (0.73–1.36)
Per 10 μg/L	0.95 (0.87–1.04)	1.04 (0.96–1.14)	1.05 (0.95–1.17)	1.01 (0.91–1.11)
Asian (*n* = 233)				
< 40	1.00	1.00	1.00	1.00
40 to < 60	0.94 (0.65–1.34)	0.65 (0.42–1.02)	0.80 (0.49–1.29)	0.89 (0.62–1.26)
≥ 60	0.77 (0.49–1.22)	0.72 (0.44–1.20)	0.68 (0.35–1.29)	0.86 (0.54–1.37)
Per 10 μg/L	0.90 (0.79–1.03)	0.95 (0.82–1.09)	0.88 (0.74–1.04)	0.86 (0.74–1.01)

a HRs were adjusted for infant sex, marital status, adequacy of prenatal care, maternal age, maternal race/ethnicity, maternal education, parity, maternal smoking, prenatal care source of payment, conception season, birth season, per-capita income, previous preterm or SGA infant, previous trimester TTHM exposure, and the presence of one or more maternal disease factors including lung disease, diabetes, eclampsia, hydramnios, chronic hypertension, pregnancy-related hypertension, incompetent cervix, renal disease, uterine bleeding, and inappropriate maternal weight gain/loss.

**Table 4:** Crude and adjusted^a^ HRs (95% CIs) for preterm birth and gestational age specific TTHM exposure averages by payment source for prenatal care.

	2nd Trimester		4 Weeks before birth	
TTHM (μg/L)	Government^b^ (*n* = 1,129)	Private^c^ (*n* = 1,684)	Government^b^ (*n* = 1,129)	Private^c^ (*n* = 1,684)
Crude				
< 40	1.00	1.00	1.00	1.00
40 to < 60	0.88 (0.75–1.02)	0.92 (0.81–1.04)	1.01 (0.84–1.23)	0.92 (0.79–1.08)
≥ 60	0.84 (0.71–0.99)	0.88 (0.77–1.01)	1.19 (0.98–1.44)	0.94 (0.80–1.10)
Adjusted				
< 40	1.00	1.00	1.00	1.00
40 to < 60	0.88 (0.72–1.07)	0.87 (0.74–1.03)	1.07 (0.85–1.34)	0.96 (0.80–1.15)
≥ 60	0.83 (0.67–1.04)	0.82 (0.69–0.99)	1.39 (1.06–1.81)	1.00 (0.79–1.25)
Per 10 μg/L	0.96 (0.91–1.02)	0.95 (0.90–1.00)	1.03 (0.96–1.11)	1.00 (0.95–1.06)

a HRs were adjusted for infant sex, marital status, adequacy of prenatal care, maternal age, maternal race and ancestry, maternal education, parity, maternal smoking, conception season, birth season, per-capita income, previous preterm or SGA infant, previous-trimester TTHM exposure, and the presence of one or more maternal disease factors including lung disease, diabetes, eclampsia, hydramnios, chronic hypertension, pregnancy-related hypertension, incompetent cervix, renal disease, uterine bleeding, and inappropriate maternal weight gain/loss.

b Source of payment was government or Healthy Start.

c Source of payment was private or health maintenance organization.
